# Editorial: Biocompatibility of implanted devices, modulation, and repair in the nervous system

**DOI:** 10.3389/fnins.2024.1505912

**Published:** 2024-10-28

**Authors:** Michael B. Christensen, Xinyan T. Cui, Loren Rieth, David J. Warren

**Affiliations:** ^1^Division of Urology, Department of Surgery, University of Utah School of Medicine, Salt Lake City, UT, United States; ^2^Department of Otolaryngology—Head and Neck Surgery, University of Utah School of Medicine, Salt Lake City, UT, United States; ^3^Department of Bioengineering, University of Pittsburgh, Pittsburgh, PA, United States; ^4^Department of Mechanical and Aerospace Engineering, West Virginia University, Morgantown, WV, United States; ^5^Feinstein Institute for Medical Research, Manhasset, NY, United States; ^6^Department of Biomedical Engineering, University of Utah, Salt Lake City, UT, United States

**Keywords:** microelectrode, foreign body response (FBR), inflammation, modulation, repair

The complexity of the nervous system poses both significant challenges and opportunities for research aimed at modulating function, repairing damage, and integrating with neural signals. Neural implants, such as brain-machine interfaces (BMIs), hold tremendous potential for restoring lost function in patients with neurological injuries or diseases. These technologies, however, must overcome the inherent challenges of biocompatibility and long-term stability within the delicate environment of the nervous system. Research in these areas has been ongoing for decades, yet the field continues to evolve rapidly, driven by advancements in materials science, neural modulation techniques, and our deepening understanding of the brain's repair mechanisms (Lebedev and Nicolelis, 2017; Seymour and Kipke, 2007).

Modulating neural activity and promoting neural repair have been longstanding goals in neuroscience, dating back to early efforts in electrical stimulation of the brain. As our understanding of the neurobiology of repair grows, researchers are discovering new ways to interface with the nervous system using electrodes, sensors, and biomaterials designed to minimize adverse immune responses while maintaining signal integrity (Panuccio et al., 2018; Shi et al., 2021). Biocompatibility, in particular, remains a critical focus. Foreign body responses (FBR), tissue damage, inflammatory gliosis, and neural cell loss are among the key challenges to creating implants that can perform reliably over years or even decades (Polikov et al., 2005; Prasad and Sanchez, 2012; Kozai et al., 2015).

Recent advances in biomaterials and implant design have led to more sophisticated devices capable of modulating and repairing neural circuits. These developments promise to enhance the integration of prosthetic devices and neural interfaces in ways that are both functional and durable. The articles within this Research Topic exemplify this progress, offering insights into how neural implants interact with tissue over time and how these interactions can be optimized for better performance and biocompatibility.

One of the critical challenges in the field of brain-machine interfaces is ensuring the long-term functionality of implanted neural electrodes. In the article *Histological confirmation of myelinated neural filaments within the tip of the neurotrophic electrode after a decade of neural recordings* by Gearing and Kennedy, the authors present a landmark study demonstrating successful, decade-long neural recordings, providing valuable insights into the biocompatibility and durability of neural implants.

The study highlights the key innovation of neurotrophic electrodes, which are designed to encourage the growth of neural tissue into the electrode's hollow tip. This design not only minimizes strain but also prevents signal loss over time—a problem commonly observed with other electrode types. The study confirmed the presence of myelinated neural filaments within the electrode and the absence of gliosis, showcasing the potential for long-term brain-machine interfaces to provide stable neural recordings without significant immune response or tissue rejection. These findings underscore the importance of integrating neural tissue into the electrode design for optimal long-term performance in clinical settings.

The second article, *Structural changes in the retina after implantation of subretinal three-dimensional implants in mini pigs* by Vu et al., addresses biocompatibility in a different context, focusing on retinal implants. The study investigates how the design of subretinal implants, specifically their geometry and size, impacts the retina's structural integrity over time. Using mini pig models, the researchers evaluated three types of implants and found that those with sloped edges and lower electrode heights preserved retinal structure more effectively than thicker, right-angled designs.

This work emphasizes the significance of implant design in ensuring long-term compatibility with delicate tissues such as the retina. The findings demonstrate that minimizing implant height and using sloped edges reduce physical stress on the retinal layers, leading to better integration and fewer adverse reactions such as fibrosis. These insights have implications for the design of future retinal prosthetics and other neural implants, where both mechanical and biological factors must be considered to optimize performance and biocompatibility.

In the article *Layer-dependent stability of intracortical recordings and neuronal cell loss* by Urdaneta et al., the authors explore how the depth of implanted electrodes within the cortex affects both recording stability and tissue health. The study demonstrates that electrodes positioned in deeper cortical layers (L4–L5) exhibited the highest long-term stability in terms of spike amplitude and signal-to-noise ratio. Using a novel machine learning-guided histological technique, the authors also revealed that neuronal cell loss was most significant in the upper cortical layers (L2/3 and L4).

This study is particularly valuable for the design of intracortical neuroprostheses, as it highlights the importance of electrode placement within the cortical architecture. By identifying the layers that are most conducive to long-term recording stability, the findings provide a foundation for optimizing electrode designs to improve performance and minimize the foreign body response.

As the population ages, the use of neural prosthetics in older patients becomes increasingly relevant. The final article, *Advanced age is not a barrier to chronic intracortical single-unit recording in rat cortex* by Nolta et al., addresses this issue by investigating the performance of intracortical implants in aged rats, a model for middle-aged humans. Despite concerns that aging might negatively affect implant performance, the study found that recording stability in older rats was comparable to that of younger rats (Black et al., 2018; Nolta et al., 2015).

The foreign body response in aged rats was also similar to younger cohorts, and the study found no significant difference in biomarkers of inflammation or tissue damage near the implant sites. These findings suggest that age alone is not a barrier to the long-term use of neural implants, offering hope for their application in older patients suffering from neurological disorders. Additionally, the study highlights the importance of minimizing vascular damage during implantation to preserve neural tissue and improve recording performance, regardless of the patient's age.

Taken together, these studies provide important advancements in our understanding of how neural implants interact with the nervous system, from the brain to the retina. The findings highlight key factors such as implant geometry, electrode depth, and patient age which influence biocompatibility, signal stability, and long-term performance. As the field continues to evolve, the insights gained from these studies will guide the design of future brain-machine interfaces, ensuring they remain functional, durable, and safe for a wide range of clinical applications. Continued research will be essential for overcoming the remaining challenges in integrating technology with the nervous system, ultimately improving the quality of life for patients with neurological impairments.
